# Serum Containing Tao-Hong-Si-Wu Decoction Induces Human Endothelial Cell VEGF Production via PI3K/Akt-eNOS Signaling

**DOI:** 10.1155/2013/195158

**Published:** 2013-05-21

**Authors:** DengKe Yin, ZhuQing Liu, DaiYin Peng, Ye Yang, XiangDong Gao, Fan Xu, Lan Han

**Affiliations:** ^1^School of Pharmacy, Anhui University of Traditional Chinese Medicine, Hefei 230031, China; ^2^State Key Laboratory of Natural Medicines, China Pharmaceutical University, Nanjing 210009, China; ^3^Anhui Provincial Key Laboratory for Chinese Medicine Research and Development, Hefei 230038, China

## Abstract

Tao-Hong-Si-Wu decoction (TSD) is a famous traditional Chinese medicine (TCM) and widely used for ischemic disease in China. TSD medicated serum was prepared after oral administration of TSD (1.6 g/kg) twice a day for 3 days in rats. TSD medicated serum induced human umbilical vein endothelial cells (HUVECs) proliferation, VEGF secretion, and nitric oxide (NO) production. These promoted effects of TSD were partly inhibited by treatment with PI3K inhibitor (LY294002) or eNOS inhibitor (L-NAME), respectively, and completely inhibited by treatment with LY294002 and L-NAME simultaneously. Western blot analysis findings further indicated that TSD medicated serum upregulated p-Akt and p-eNOS expressions, which were significantly inhibited by LY294002 or L-NAME and completely inhibited by both LY294002 and L-NAME; these results indicated that TSD medicated serum induced HUVECs VEGF expression via PI3K/Akt-eNOS signaling. TSD medicated serum contains hydroxysafflor yellow A, ferulic acid, and ligustilide detected by UPLC with standards, so these effect of TSD medicated serum may be associated with these three active compounds absorbed in serum.

## 1. Introduction

Ischemic diseases, especially ischemic heart disease, remain a major and well-researched challenge for humans. In the process of angiogenesis, modulation of endothelial cells plays a key role in such processes as proliferation, migration, and assembly. Numerous regulatory angiogenic factors have been identified, and their molecular modulations have been associated with several angiogenic disorders [1, 2]. Therapeutic angiogenesis is the clinical use of methods to enhance or promote the development of collateral blood vessels in ischemic tissue and is critical to ischemic diseases such as myocardial infarction and stroke. Angiogenesis is the formation of new blood vessels from preexisting capillaries in embryonic development, wound healing, and cardiovascular disease [3]. Although increasing evidence indicates that angiogenesis is a highly sophisticated and coordinated process, the activation of endothelial cells and release of angiogenic factors are the most important steps. The survey and development of new agents promoting angiogenesis via growth factors have become a focus of therapeutic strategies for these ischemic diseases [4].

Tao-Hong-Si-Wu decoction (TSD) is a famous traditional Chinese medicine, first recorded in Yizong jinjian (Golden Mirror of Medicine, 1749) by Wu Qian, and widely used for blood stasis syndrome with a history of several centuries. The formula mainly consists of six plant materials (Table 1). Traditional Chinese medicine practitioners described the function of TSD as “promoting blood circulation to remove blood stasis.” In clinical practice, TSD could open the blood vessels and promote blood flow in circulation to relive woman's irregular menses disorder and is also used to treat cardiovascular diseases such as hypertension and angina. Furthermore, it can increase blood flow of the microcirculation thereby regulating diabetic neuropathies and glucocorticoid-induced avascular necrosis of the femoral head [5].

Many researchers believed that serum pharmacology is more scientific and more befitting for Chinese traditional medicine than traditional pharmacology in which crude drugs are directly added into the culture system of cells or organs in vitro [6, 7]. Medicine or medicine compounds are orally administered to animal, blood is collected to separate to the serum after a definitive period of time, and the drug serum is ready for experimental analysis in vitro. Although TSD has been widely used in ischemic disease, the effects of TSD on the critical step of angiogenesis, endothelial cell activation, has not been clarified. The aim of this study is to investigate the effect of TSD on endothelial cell proliferation and release of VEGF with the method of serum pharmacology.

## 2. Materials and Methods

### 2.1. Materials

#### 2.1.1. Composition and Preparation of TSD

TSD consists of six medicinal plants as shown in Table 1. Six herbs were purchased from Hefei He Yi Tang Traditional Chinese Medicines Limited Liability Company and identified by Professor Dequn Wang in the School of Pharmacy, Anhui University of Traditional Chinese Medicine. 

TSD were prepared according to the following procedure: six medicinal materials were mixed in proportion and were macerated for 6 h with ten times (v/w) 75% ethanol. The medical solution was heated to boiling then refluxed for 1.5 h and filtrate was collected. The residue was refluxed again for 1.5 h, with eight times (v/w) 75% ethanol; then filtrate was collected again and mixed with previous collected filtrate and condensed and dried at 65°C. The yield of dried powder was 18.27% according to the original herbs. The doses were presented as such powder suspended in the distilled water. 

#### 2.1.2. Reagent

 Other drugs and reagents used in this study are as follows: Akt, p-Akt, and p-eNOS antibody were purchased from Abzoom biolabs, Inc., import packing. Anti-PIP3 antibody was purchased from Echelon Biosciences. LY294002 was purchased from Gibco Company, and L-NAME was purchased from Beijing Grandsky Company. The VEGF and Ang-1 kits were purchased from the R&D Company, and the NO kit was purchased from Nanjing Jiancheng Bioengineering Institute. Reagents and chemicals standard for ultrahigh performance liquid chromatography (UPLC) detection: CH_3_CN and H_3_PO_4_: TEDIA (Fairfield, OH, USA); gallic acid, paeoniflorin, ferulic acid, tetramethylpyrazine, ligustilide, and hydroxysafflor yellow A were purchased from the National Institute for the Control of Pharmaceutical and Biological Products (Beijing, China).

#### 2.1.3. Animals

Twenty-eight clean healthy Sprague-Dawley rats (all females), weighing 200–220 g, were provided by the Laboratory Animal Center of Anhui Medical University and housed in a cage at 23 ± 1°C with a 12-hour light-dark cycle. Food and water were freely available. The protocol was performed in accordance with the Guidance Suggestions for the Care and Use of Laboratory Animals (National Research Council of USA, 1996) and related ethical regulations of our university.

#### 2.1.4. Umbilical Cord

Healthy sterile umbilical cords were provided by Obstetrics and Gynecology of Anhui Provincial Maternal and Child Health Hospital and Obstetrics and Gynecology of First Affiliated Hospital, Anhui Medical University.

### 2.2. Methods

#### 2.2.1. Cell Culture

HUVECs were isolated from healthy umbilical cords obtained through local hospitals under approval of the appropriate institutional review board (IRB). The process was as follows: untraumatized umbilical cord segments, 15–20 cm in length, the umbilical vein was cannulated and perfused with Dulbecco's phosphate-buffered aline (PBS, with calcium and magnesium) to remove all traces of blood; the vein lumen was filled with 1 mg/mL collagenase I; after a 18 min incubation at 37°C, the contents of the vein were gently flushed out with an equal volume of M199 supplemented with 20% fetal bovine serum (FBS) (Hyclone) and centrifuged at 1000 r/min for 10 minutes, and cells were resuspended in M199 (containing 20% FBS, penicillin 100 ku/L streptomycin 100 ku/L, basic fibroblast growth factor (bFGF) (PeproTech Inc.) 10 ng/mL, L-glutamine 2 mmol/L), packaging into 1% gelatin-coated flasks for culturing and incubating at 37°C and 5% CO_2_. After 24 h, the medium was changed to remove residual blood cells and nonadherent cells, the medium was changed every 2-3 days, and cultured flasks were covered by cells.

#### 2.2.2. Preparation of TSD Medicated Serum

The 28 Sprague-Dawley rats were randomly divided into TSD (*n* = 14) and blank control (*n* = 14) groups. Rats in the TSD group received intragastric administration of TSD (1.6 g/kg) twice a day for 3 days. The control group received intragastric injection of physiological saline twice a day for 3 days. One hour after the last administration, rats were intraperitoneally anesthetized by amobarbital and blood was sampled from the abdominal aorta and centrifuged. The serum was aliquoted into 10 mL ampoules and preserved at −80°C for future use.

#### 2.2.3. Plasma Samples Preparation for UPLC Analysis

 To tube containing 2 mL plasma, 6 mL methanol were added, and mixture was then vortexed for 2 min. The sample was then centrifuged for 10 min at 1000 g, the supernatant were removed, and 6 mL methanol were added into precipitate then vortexed and centrifuged for collecting the supernatant, supernatant was mixed and evaporated to dryness by nitrogen gas. Methanol (200 *μ*L) was used to dissolve extraction and the filtration through a 0.2 *μ*m syring fiter of dissolved extraction as sample for UPLC analysis. Analytical UPLC was performed on a Waters UPLC, acuity H-Class system combined with a Waters Acquity quaternary solvent manager (QSM), an Acquity sample manager (SM), a column heater, a 2996 PDA detector, and an in-line degasser system; the procedure was as follows: using a Welchorm-C18 column 6 mm × 250 mm, 5 *μ*m) (Welch Materials, Inc., MD, USA), eluting with solvents (A) 0.05% (v/v) phosphoric acid and (B) acetonitrile (CH_3_CN). The separation was initiated with 85% A and 15% B for 3 min, then a linear gradient changing rate from 85% A and 15% B to 0% A and 100% B over 20 min, and then 100% B was continued for 10 min. The flow rate was controlled with LC 20AD as 0.5 *μ*L/min, and the column temperature was maintained at 30°C. The effluents from the column were detected at absorbance of 304 nm using a photodiode-array detector. 

#### 2.2.4. Evaluation of Cell Proliferation

Cells were seeded in 96-well microplates (1 × 10^4^ cells/well in 200 *μ*L medium), routinely cultured in a humidified incubator (37°C in 5% CO_2_) for 24 hours blank serum and TSD medicated serum in serial concentration (5%, 10%, 15%), and re-incubated for 24 hours in order to clarify the influence of PI3K inhibitor (LY294002) and eNOS inhibitor (L-NAME) on TSD-induced cell proliferation. Subconfluent Cells were cultured with 10% blank serum or 10% medicated serum with LY294002 (final concentration is 20*μ*M) or L-NAME (final concentration is 750 *μ*M) or not for 24 hours; then the medium was discarded, and 5 mg/mL of tetrazolium dye (MTT) solution was added to every well and reincubated for an additional 4 hours. 10 *μ*L of DMSO was added to dissolve the formazan crystals formed. The plate was then read on a microplate reader at 490 nm. The viability of the cultures was expressed as a percentage of the absorbance measured in control cells.

#### 2.2.5. Nitrite (NO^2−^)/Nitrate (NO^3−^) Assay

Cultured cells were assayed for NO synthesis by measuring the stable end product of NO, NO^2−^, and NO^3−^ according to the instruction of manual. The cells were cultured in standard cultured flasks (5–7 × 10^5^ cells). Blank serum and TSD medicated serum in serial concentrations were reincubated for 24 hours. 

#### 2.2.6. Enzyme-Linked Immunosorbent Assay (ELISA)

 VEGF in conditioned media was quantified using a commercially available enzyme-linked immunosorbent assay kit (R&D Systems). Absorbance was measured at 450 nm using a microplate reader (BMG Labtech, Offenburg, Germany). All samples were run in duplicate.

#### 2.2.7. Western Blotting

Cells were lysed with buffer containing (in mM) Tris-HCl 50, NaCl 100, MgCl_2_ 10, EDTA-2, and 0.1% Triton X-100. The cell lysates were centrifuged at 10000 r/min and the supernatant collected. The protein concentration was determined using the Bradford assay. The cell lysates (20 mg) were separated with sodium dodecyl sulfate polyacrylamide gel electrophoresis and transferred to polyvinylidene fluoride membranes. After blocking with 5% goat serum (Hangzhou Sijiqing Biological Engineering Materials Co., Ltd., Hangzhou, Zhejiang Province, China), the membranes were incubated in rabbit anti-Akt polyclonal antibody (1 : 500 dilution; Abzoom biolabs, Dallas, TX, USA), rabbit anti-phospho-Akt polyclonal antibody (1 : 500 dilution; Abzoom biolabs, Dallas, TX, USA), and mouse anti-phospho-eNOS monoclonal antibody (1 : 500 dilution; Abzoom biolabs, Dallas, TX, USA) at 4°C for one night. This was followed by incubation with horseradish peroxidase-conjugated goat antirabbit IgG (1 : 3000, Rockland, Gilbertsville, PA, USA) and goat antimouse IgG (1 : 2500, Rockland, Gilbertsville, PA, USA) at 37°C for one hour. Expression of anti-*β*-actin (1 : 500, *β*-actin antibody, Santa Cruz Biotechnology, Santa cruz, CA, USA) was used as the loading control. The band intensities were analyzed with computer software, Image J (National Institute for Health, Bethesda, MD, USA). 

#### 2.2.8. Detection of PIP3 Levels

The relative PIP3 levels in cellular were determined by flow cytometry; cells were seeded in 6-well microplates (2 × 10^5^ cells/well in 2 mL medium) and cultured for 24 hours, and then the medium was replaced by 10% blank serum, 10% medicated serum, 10% blank serum with LY294002 (20 *μ*M), and 10% medicated serum with LY294002 (20 *μ*M) for 24 h, washed with PBS and scraped slightly to collect cells, fixed in 2% paraformaldehyde, permeabilized with 0.5% saponin in PBS, and then washed and incubated overnight at 4°C with anti-PIP3 antibody (1 : 50). Washed with PBS for three times, the primary antibody was detected with a FITC conjugated secondary antibody. The FITC fluorescence was detected by FACS Calibur flow cytometer (Becton-Dickinson, San Diego, CA) equipped with multicolor analysis capability. For each sample, 10000 cells were collected.

#### 2.2.9. Statistical Analysis

Data were analyzed using SPSS 17.0 software (SPSS, Chicago, IL, USA) and presented as mean ± SD. One-way analysis of variance was used to evaluate differences between groups. The Fishers least significant difference post hoc test was used to analyze differences between groups. A value of *P* < 0.05 was considered statistically significant.

## 3. Results

### 3.1. UPLC Analysis of TSD Medicated Serum

**Figure 1:**
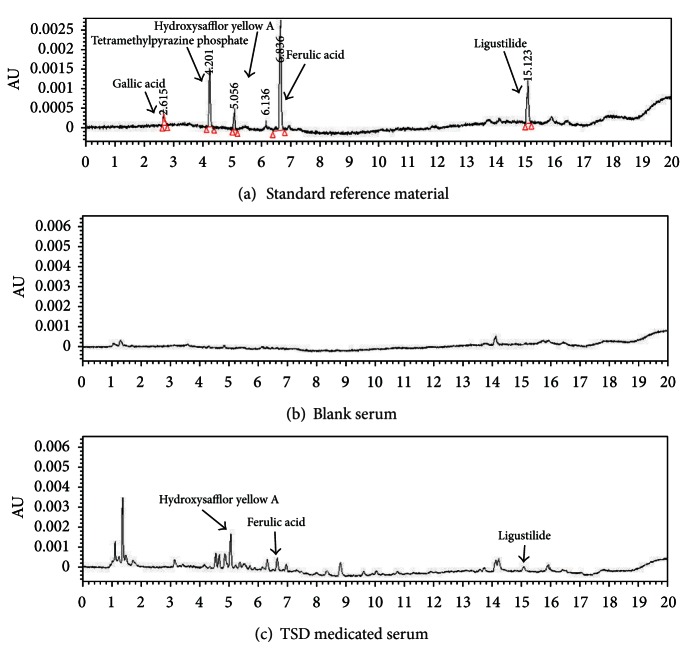
UPLC pattern of TSD medicated Serum.

The UPLC pattern of TSD medicated serum indicates its complex components with several peaks of varied retention times (Figure 1), those components including prototype component in crude drugs and its metabolites. By referring to standards, we can confirm that TSD medicated serum contains hydroxysafflor yellow A, ferulic acid, and ligustilide. 

### 3.2. TSD Medicated Serum Induced HUVECs Proliferation

**Figure 2:**
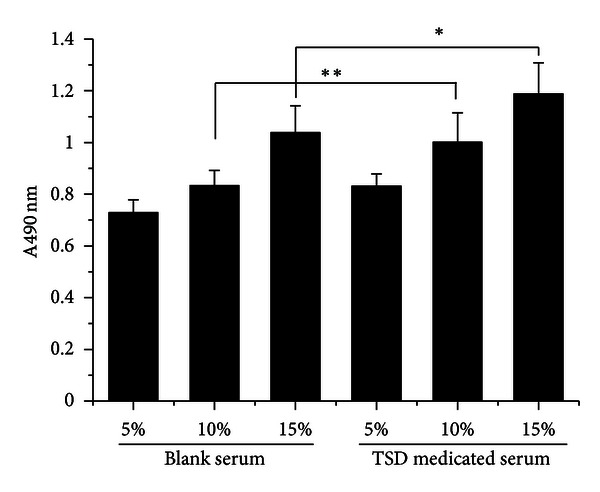
TSD medicated serum-induced HUVECs proliferation. Cells were treated with blank serum or TSD medicated serum for 24 h, and the proliferations of HUVECs were determined by MTT assay. The data were expressed as mean  ±  SD, *n* = 8. *P < 0.05 compared with 15% blank serum, **P < 0.01 compared with 10% blank serum.

**Figure 3:**
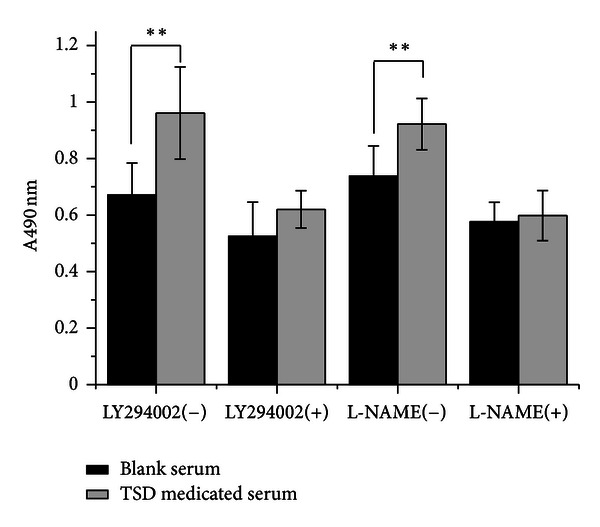
The effect of LY294002 and L-NAME on TSD medicated serum induced HUVECs proliferation. Cells were treated with 10% blank serum or 10% TSD medicated serum for 24 h in presence of LY294002 or L-NAME or not. The proliferations of HUVECs were determined by MTT assay. The data were expressed as mean  ±  SD, *n* = 8. **P < 0.01.

The MTT conversion test showed that the HUVECs proliferation was promoted by TSD medicated serum in different concentration (5%–10%) compared with the same concentration of blank serum (Figure 2); 10% TSD medicated serum significantly promoted HUVECs proliferation compared with 10% blank serum on cell proliferation (*P* < 0.01). However, the promoted effect on HUVECs proliferation were significantly inhibited by LY294002 (20 *μ*M) or L-NAME (750 *μ*M) (Figure 3).

### 3.3. TSD Medicated Serum Induced NO Production in HUVECs

**Figure 4:**
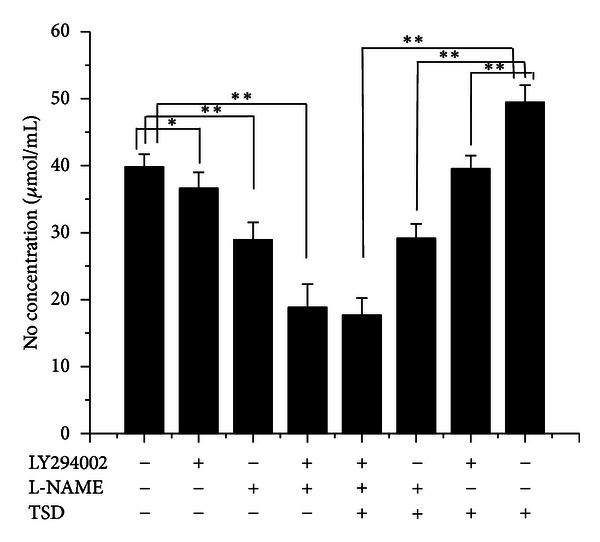
The effect of LY294002 and L-NAME on TSD medicated serum induced NO production in HUVECs. Cells were treated with 10% blank serum or 10% TSD medicated serum for 24 h in presence of LY294002 or L-NAME or not; the production of NO was determined by measuring the stable end product of NO, NO^2−^, and NO^3−^. The data were expressed as mean ± SD, *n* = 8. *P < 0.05 and **P < 0.01.

Supernatants were used to detect the TSD medicated serum on NO production. Compared with the same concentration of the blank serum, NO production was promoted by TSD medicated serum (concentration of 10% serum). While LY294002 (20 *μ*M) or L-NAME (750 *μ*M) was incubated with endothelial cells could significantly inhibit TSD medicated serum induced NO production, there was no significantly difference between group of blank serum with two inhibitors and TSD medicated serum with two inhibitors (*P* > 0.05); these results indicated that the production of NO promoted by TSD medicated serum was completely inhibited by two inhibitors coincubated with HUVECs (Figure 4).

### 3.4. TSD Medicated Serum Induced Expression of VEGF in HUVECs

**Figure 5:**
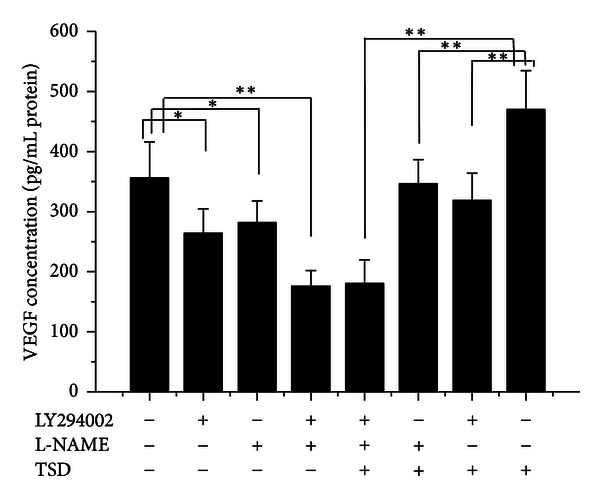
The effect of LY294002 and L-NAME on TSD medicated serum induced VEGF production in HUVECs. Cells were treated with 10% blank serum or 10% TSD medicated serum for 24 h in presence of LY294002 or L-NAME or not; the production of NO was determined by ELISA according to the manual. The data were expressed as mean ± SD, *n* = 8. *P < 0.05 and **P < 0.01.

VEGF is known to be a key activator of angiogenesis. In order to clarify the effect of TSD medicated serum on expression of VEGF in HUVECs, the supernatant in conditioned medium was determined by ELISA. Treatment with TSD medicated serum (concentration of 10% serum) could promote the expression of VEGF in HUVECs compared with HUVECs treatment with 10% blank serum; while these promoted effect of TSD medicated serum was significantly inhibited by LY294002 (20 *μ*M) or L-NAME (750 *μ*M), especially, when the two inhibitors coincubated with HUVECs, the upregulation of TSD medicated serum was completely inhibited (Figure 5).

### 3.5. TSD Medicated Serum Induced the Expression of p-Akt and p-eNOS in PI3K/Akt-eNOS Pathways

**Figure 6:**
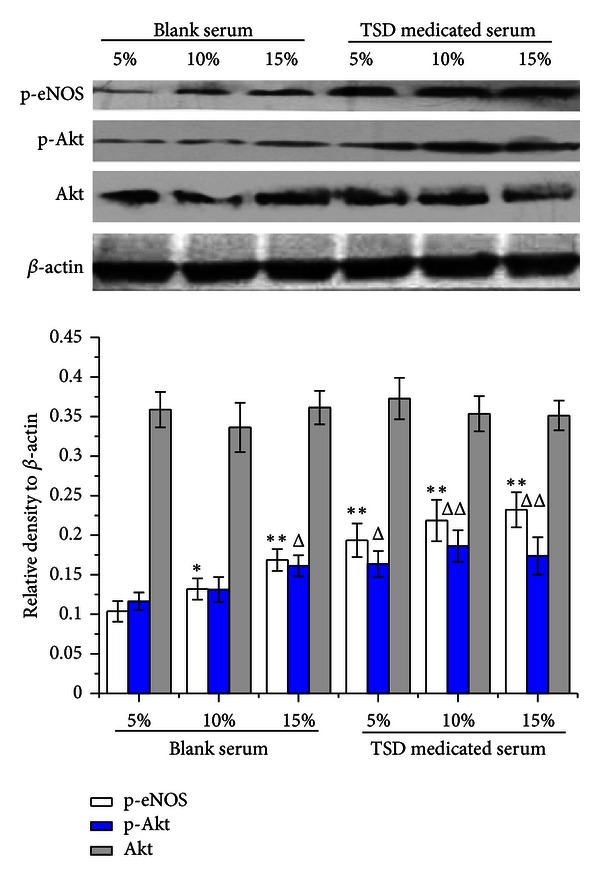
Effects of different concentrations of TSD medicated serum on expression of p-Akt, total Akt and p-eNOS. Cells were treated with blank serum (5–10%) or TSD medicated serum (5–10%) for 24 h. Cells were lysed, and the expression was determined by western blot with specific primary antibody to p-Akt, total Akt, and p-eNOS; bands were displayed with secondary antibody and ECL system. Data were expressed as mean ± SD, *n* = 3. *P < 0.05 and **P < 0.01 compared with p-eNOS of 5% blank serum group, ^Δ^ *P* < 0.05 and ^ΔΔ^ *P* < 0.01 compare with p-Akt of 5% blank serum group.

**Figure 7:**
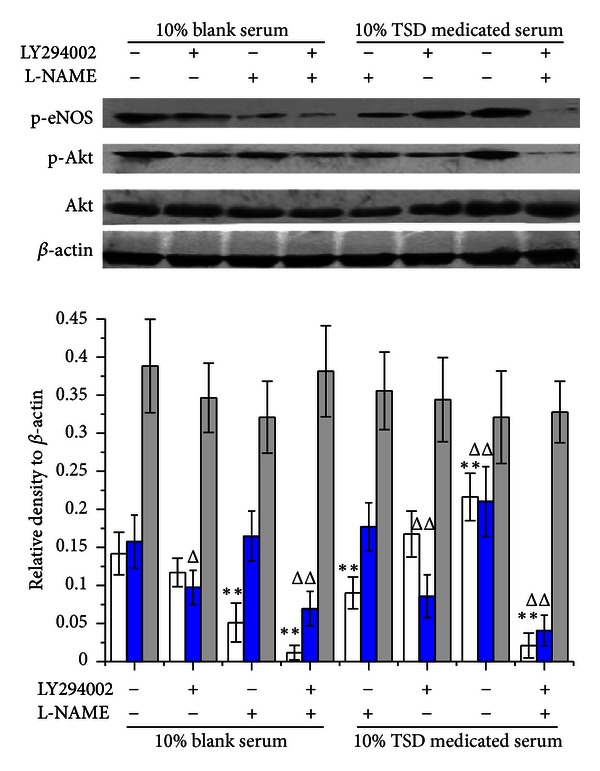
Effects of 10% TSD medicated serum on expression of p-Akt, total Akt, and p-eNOS in presence of LY294002 and L-NAME. Cells were treated with 10% blank serum or 10% TSD medicated serum for 24 h in presence of LY294002, L-NAME, or both. Cells were lysed, and the expression was determined by western blot with specific primary antibody to p-Akt, total Akt, and p-eNOS; bands were displayed with secondary antibody and ECL system. Data were expressed as mean ± SD, *n* = 3. *P < 0.05 and **P < 0.01 compared with p-eNOS of 10% blank serum group, ^Δ^ *P* < 0.05 and ^ΔΔ^ *P* < 0.01 compare with p-Akt of 10% blank serum group.

With the concentration of serum increased, the protein expression of p-Akt and p-eNOS was increased in HUVECs treated with blank serum or TSD medicated serum, while total Akt expression did not significantly changed (Figure 6). There was more expression of p-Akt and p-eNOS in TSD medicated serum than the same serum concentration of blank serum (Figure 6). LY294002 (20 *μ*M) significantly decreased the expression of p-Akt in HUVECs compared with groups without inhibitor, as well as L-NAME (750 *μ*M) decreased the expression of p-eNOS (Figure 7). The expression of p-Akt and p-eNOS in groups of 10% TSD medicated serum without inhibitors was much more than in groups of 10% blank serum without inhibitors. While in presence of LY294002 and L-NAME, the upregulated effect of medicated serum on expression of p-Akt and p-eNOS was completely inhibited (Figure 7). 

### 3.6. TSD Medicated Serum Enhanced the Level of PIP3

**Figure 8:**
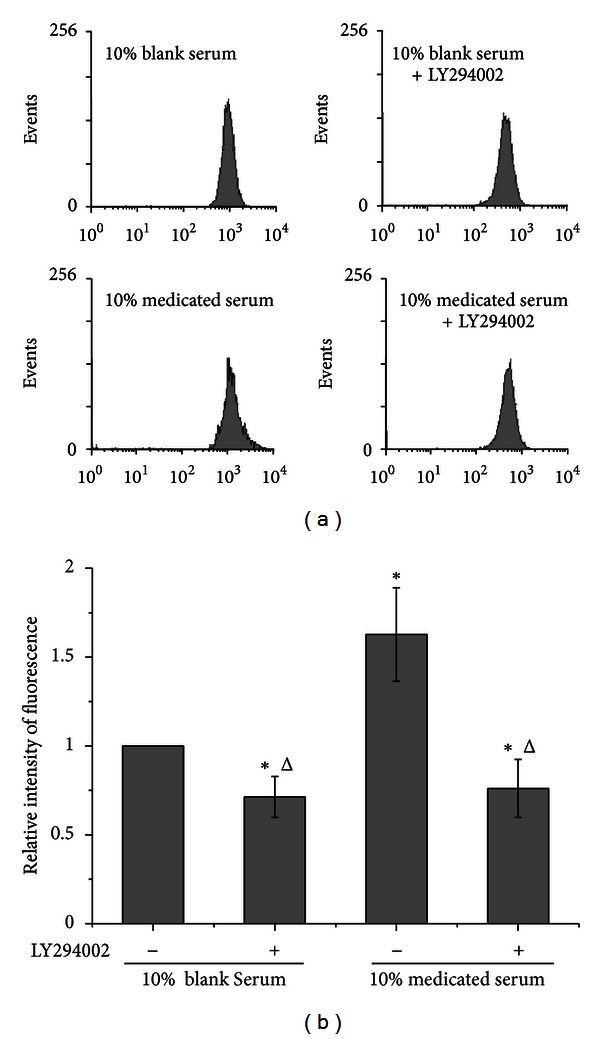
Effects of 10% TSD medicated serum on the levels of PIP3. Cells were treated with 10% blank serum or 10% TSD medicated serum for 24 h in presence of LY294002 or not. The levels of PIP3 were analysis by flow cytometry with anti-PIP3 antibody and FITC conjugated secondary antibody. (a) is the representative figure of flow cytometry. (b) is the bar graph of relative intensity of fluorescence in three independent experiments. Data were expressed as mean ± SD. *P < 0.05 compared with 10% blank serum and ^Δ^ *P* < 0.05 compared with 10% medicated serum.

 In PI3K/Akt signaling, PIP2 activated PIP3 by PI3K, which caused Akt activation. In order to clarify the effect of TSD medicated serum on PI3K, the level of PIP3 was analyszed by flow cytometry with anti-PIP3 antibody. 10% TSD medicated serum enhanced the level of PIP3 in HUVECs compared with 10% blank serum-treated HUVECs; the enhanced level of PIP3 was completely inhibited by PI3K inhibitor (LY294002) (Figure 8). 

## 4. Discussion 

Cardiovascular disease is the leading cause of death worldwide and is often associated with partial or full occlusion of the blood vessel network in the affected organs. Restoring blood supply is critical for the successful treatment of cardiovascular disease [8]. Endothelial cell dysfunction is the main reason for diverse cardiovascular disease; insufficient angiogenesis is caused by the inadequate production of angiogenesis growth factors and/or excessive amounts of angiogenesis inhibitors [9]. Angiogenic factors bind to their receptors on endothelial cells, and promoting endothelial cells proliferation is the critical step in angiogenesis. Vascular endothelial growth factor (VEGF) is now known as a multifunctional peptide capable of inducing receptor-mediated endothelial cell proliferation and angiogenesis both in vivo and in vitro. It has a crucial role in embryonic vascular development and in adult pathophysiology [10, 11].

Direct activation of the angiogenic signal pathways and production of angiogenic factors including VEGF increase neovascularization [12]. VEGF promote angiogenesis by activating multiple signal pathways, such as the MEK/ERK pathway for endothelial cell proliferation, the PI3K/Akt/eNOS pathway for endothelial cell survival, and p125^FAK^/Src/p38 MAPK signaling system for endothelial cell migration [12, 13]. Our data showed that TSD medicated serum promotes the expression of VEGF in HUVECs, indicating that TSD may indirectly activate the angiogenic signal pathways. Indeed, we confirmed that TSD medicated serum induced the proliferation of HUVECs by PI3K/Akt-eNOS dependent pathways, which are essential for the activation of endothelial cells induced by direct angiogenic factors [12].

Endothelial dysfunction characterized by a decrease in the bioavailability of vasodilator like a nitric oxide (NO) has been observed in individuals with vascular complications [14]. In particular, endothelium-derived NO is a cerebrovascular protector and an important endogenous mediator of vascular homeostasis and blood flow [15]. The loss of endothelial NO impairs vascular function, in part by promoting vasoconstriction, platelet aggregation, smooth muscle cell proliferation, and leukocyte adhesion [16, 17]. In endothelial cells, NO is synthesized from the substrate L-arginine via eNOS, and the phosphorylation of a specific serine residue (Ser-1177) in eNOS is important for its enzymatic activity [18]. VEGF is known to induce the release of NO from endothelial cells, and vascular endothelium inducible NO synthase production is amplified during VEGF-induced angiogenesis [19]. The role of NO in VEGF-induced angiogenesis has been shown in NOS knocked out mice as well as after NOS inhibition, both result in reduction of angiogenesis [20–22]. NO has also a regulatory effect on VEGF production [23–25].

In these experiment, TSD medicated serum increased the expression of VEGF in HUVECs as well as production of NO. It have been demonstrated that VEGF enhances the expression of eNOS in native and cultured endothelial cells, an effect that may be important in the process of VEGF-induced angiogenesis [21, 26]. Inhibitors of NOS suppress angiogenesis, and the proliferative effect of VEGF is decreased in the presence of NOS inhibitor [27]. Our results indicated that PI3K inhibitor suppressed the promoted effect of TSD medicated serum on expression of VEGF and production of NO, as well as eNOS inhibitor decreased the expression of VEGF and production of NO. Simultaneously treated with PI3K inhibitor and eNOS inhibitor, the effect of TSD medicated serum on VEGF expression and NO production of endothelial cell was completely inhibited, this also proved that TSD medicated serum activated the HUVECs by PI3K/Akt-eNOS pathways. In order to obtain the direct evidence on PI3K/Akt-eNOS pathways, the effect of TSD medicated serum on expression of p-eNOS, p-Akt and Akt was investigated by western blot. The expression of p-eNOS and p-Akt was significantly increased in groups of TSD medicated serum compared with blank serum groups, but had no significant influence on Akt expression. PI3K is the upstream molecular in PI3K/Akt-eNOS pathway, and PI3K activation could catalyze the PIP2 to PIP3, so the level of PIP3 was used to indicated the activity of PI3K; the results also indicated that TSD medicated serum have promoted effect on PI3K. These results also clarified that TSD medicated serum has an effect on PI3K/Akt-eNOS pathways.

TSD consists of six medicinal plants, in which Danggui and Chuanxiong could affect VEGF expression in rat myocardial infarction, promote endothelial cell proliferation, and stimulate quantity of vessels on chick embryo chorioallantoic membrane (CAM) [28]. Because the active compounds of these medicinal plants should be absorbed and translated into serum, the active compounds could play their therapeutic effect. Using UPLC with standard compounds, we found that TSD medicated serum containing Hydroxysafflor yellow A (HSYA), ferulic acid, and ligustilide. Previously, studies indicated that HSYA could protect HUVECs from hypoxia-induced injuries [29], enhance the survival of endothelial cell, and induce the express of VEGF [30]. HSYA also has a therapeutic effect on focal cerebral ischaemia in vivo [31, 32]; HSYA protects the myocardium against ischaemia-reperfusion injury through enhanced nitric oxide production by eNOS activation [33]. Ferulic acid promotes endothelial cells proliferation through upregulation VEGF [34] and augments angiogenesis [35]. Ligustilide also has protective effects against ischaemia-reperfusion [36] and alleviates brain damage of chronic cerebral hypoperfusion [37]. So we could speculate that the effect of TSD on endothelial cell is mainly associated with these three active compounds absorbed in serum.

## Figures and Tables

**Table 1:** The recipe of Tao-Hong-Siwu-Tang (TSD).

Components	Ratio
Shu Di Huang (*Rehmannia glutinosa *Libosch)	4
Bai Shao (*Paeonia lactiflora *Pallas)	3
Dang Gui (*Angelica sinensis* (Oliv.) Diels)	3
Chuan Xiong (*Ligusticum chuanxiong* Hort.)	2
Tao Ren (*Prunus persica* (L.))	3
Hong Hua (*Carthamus tinctorius* L)	2
